# Transthoracic Echocardiography in Children and Young Adults with Congenital Heart Disease

**DOI:** 10.5402/2012/753481

**Published:** 2012-06-13

**Authors:** Martin Koestenberger

**Affiliations:** Division of Pediatric Cardiology, Department of Pediatrics, Medical University of Graz, Auenbruggerplatz 30, 8036 Graz, Austria

## Abstract

Transthoracic echocardiography (TTE) is the first-line tool for diagnosis and followup of pediatric and young adult patients with congenital heart disease (CHD). Appropriate use of TTE can reduce the need for more invasive modalities, such as cardiac catheterization and cardiac magnetic resonance imaging. New echocardiographic techniques have emerged more recently: tissue Doppler imaging, tissue tracking (strain and strain rate), vector velocity imaging (VVI), myocardial performance index, myocardial acceleration during isovolumic acceleration (IVA), the ratio of systolic to diastolic duration (S/D ratio), and two dimensional measurements of systolic right ventricular (RV) function (e.g., tricuspid annular plane systolic excursion, TAPSE). These may become valuable indicators of ventricular performance, compliance, and disease progression. In addition, three-dimensional (3D) echocardiography when performed for the assessment of valvular function, device position, and ventricular volumes is being integrated into routine clinical care. In this paper, the potential use and limitations of these new echocardiographic techniques in patients with CHD are discussed. A particular focus is on the echocardiographic assessment of right ventricular (RV) function in conditions associated with increased right ventricular volume (e.g., pulmonary regurgitation after tetralogy of Fallot repair) or pressure (e.g., pulmonary hypertension) in children and young adults.

## 1. Introduction

Echocardiography has become the most important and routinely applied noninvasive imaging technique for the diagnosis and followup of patients with congenital heart disease (CHD). Cross-sectional Doppler echocardiography allows a detailed description of cardiovascular anatomy, ventricular, and valvular function. The diagnostic accuracy for describing cardiac morphology is very high, with a reported incidence of less than 100 errors in more than 50.000 echocardiograms [1]. Most functional variables used in echocardiography were developed and validated for the assessment of the normal, systemic, morphologically left ventricle (LV). The heterogeneity of CHD, anatomic normal variants, effects of the child's growth, and interstudy variability of hemodynamics complicate the proper interpretation of many functional variables. For the LV, adult techniques are often extrapolated to pediatrics without comprehensive validation in a large pediatric cohort or even blinded prospective studies. For the RV, qualitative (subjective) assessment is the technique used routinely in most laboratories (eye balling). Right ventricular hemodynamic function is physiologically different than that of the LV, including different RV versus LV myocardial fiber arrangements [2–4], lower RV afterload (pulmonary vascular resistance), and lower systolic RV pressures compared with the LV.

Traditionally, dimensional changes or volumetric changes during systole of the cardiac cycle have been used to assess systolic ventricular function. This can be done using M-mode techniques to calculate fractional shortening or by volumetric techniques to calculate ejection fraction. These techniques are especially load-sensitive (preload dependent) and rely on geometrical assumptions. Therefore, they may not be readily applicable to congenital heart disease. Recent technical developments have renewed the interest in the assessment of ventricular performance in patients with CHD.

Newer techniques including tissue Doppler echocardiography, tissue tracking (speckle-tracking-based strain imaging), and vector velocity imaging (VVI) provide direct quantitative information about myocardial motion and deformation. Three-dimensional (3D) echocardiographic techniques enable the acquisition of full volumetric datasets, which can be analysed offline for the calculation of ventricular volumes, mass, and EF.

**Figure 1:**
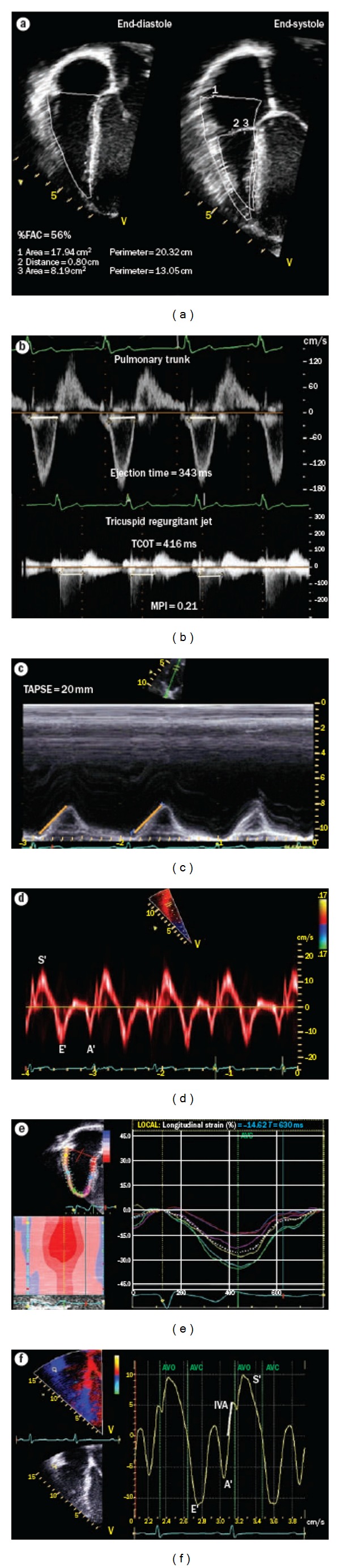
Assessment of right ventricular (RV) function by echocardiography. (a) %FAC, calculated from measures from the apical four-chamber view. The importance of longitudinal shortening can be appreciated in this image. (b) MPi, calculated by measuring the ejection time on the pulmonary artery tracing and the time between closure and opening of the tricuspid valve on the tricuspid inflow tracing. MPi = (TCOT-eT)/eT, MPi was normal after tetralogy of Fallot repair in this patient. (c) TAPSE. An M-mode echocardiogram through the tricuspid annulus is obtained, and the excursion of the tricuspid annulus is measured as illustrated. This index enables assessment of longitudinal RV function. (d) Tissue Doppler velocities of the tricuspid annulus. Pulsed tissue Doppler measurements can be used to calculate tissue velocities. Systolic velocities can be used as a parameter for systolic longitudinal RV function. (e) Longitudinal strain measurements of the right ventricle, made using speckle tracking technology. By convention, systolic longitudinal shortening is represented as a negative value and can be measured in six different segments. The mean values of these segments are used to trace a mean longitudinal strain curve (white dotted line). The value at end-systole is then measured. (f) Color tissue Doppler echocardiogram at the lateral tricuspid valve annulus and measurement of iVA. Aortic valve opening and closure are depicted by green lines for event timing. The timing of these events may be taken as that of pulmonary valve opening and closure. The slope of iVA is shown. Note that iVA occurs within the QRS complex and peaks before pulmonary valve opening in the isovolumic period. A′: late diastolic tissue velocity; AVC: aortic valve closure; AVO: aortic valve opening; e′: early-diastolic tissue velocity; eT: ejection time; %FAC: percentage fractional area change; IVA: isovolumic acceleration; MPI: myocardial performance index; S′: systolic tissue velocity; TAPSE: tricuspid annular systolic plane excursion; TCOT: tricuspid valve closure-opening time. Copyright from Mertens et al., Nat. Rev. Cardiol 2010 (doi:10.1038/nrccardio.2010.118) with permission from the publisher, Macmillan Publishers Limited, Copyright approval from Nature Publishing Group.

Transthoracic echocardiography (TTE) has the advantage of lower cost, less risk, and greater availability compared with cardiac magnetic resonance imaging or cardiac catheterization. In this paper we discuss and highlight recently developed echocardiographic techniques and according variables such as tissue Doppler imaging, tissue tracking and vector velocity imaging (deformation imaging for strain and strain rate), myocardial performance index, myocardial acceleration during IVA, systolic to diastolic ratio. Also measurements of longitudinal systolic RV function such as tricuspid annular plane systolic excursion (TAPSE) and peak systolic velocity (TAPSV) and 3D echocardiography are discussed (Figure 1).

## 2. Tissue Doppler Velocities

**Figure 2:**
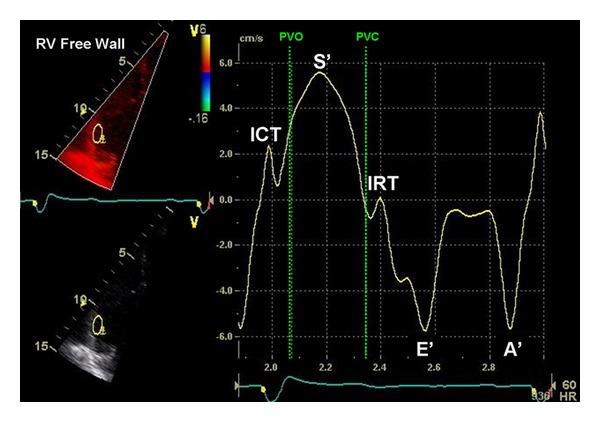
Color DTI waveforms obtained from the RV free wall describing the various phases in ventricular systole and diastole. There are 5 discrete waveforms occurring within the cardiac cycle, as seen on the Doppler display: (1) ICT velocity, (2) systolic (S0) velocity, (3) IRT velocity, (4) early diastolic velocity (E0), and (5) late diastolic velocity (A0). Dotted vertical lines represent PV opening (PVO) and PV closure (PVC). Copyright from Horton et al. (J Am Soc Echocardiogr 2009; 22: 776-792) with permission from the publisher, Copyright 2009, the Journal of the American Society of Echocardiography.

In the 1990s, tissue Doppler imaging started being recognized as a potentially clinically useful technique for the assessment of global and regional myocardial systolic and diastolic function [5]. As the myocardium moves during the contractile cycle, velocities within the myocardium can be recorded using Doppler technology [5]. By measuring these tissue Doppler velocities in different regions within the myocardium, regional myocardial function can be assessed. In patients with normal sinus rhythm, the following waveforms can be seen: the isovolumic contraction time (ICT) waveform occurs in early systole; the systolic (S) peak waveform occurs during ventricular mechanical systole and is always displayed above the zero baseline; the isovolumic relaxation time (IRT) waveform occurs in early diastole (end of the T wave on electrocardiography); the early diastolic (E) waveform occurs during peak ventricular relaxation (after IRT); the peak late diastolic (A) waveform represents atrial contraction (Figure 2).

Normal paediatric tissue Doppler imaging (TDI) data has been published [6]. It was shown that tissue velocities vary with age and heart rate. Eidem et al. showed that pulsed-wave TD velocities also correlate with cardiac growth variables (i.e., especially LV end-diastolic dimension (LVEDD) and LV mass [6]), indicating that tissue velocities are not independent of geometry. This has important implications when applying this methodology to children with congenital heart disease who have great variability in RV and LV size, mass, and geometry. Apart from the influence of geometry, changes in loading conditions also affect TDI velocity measurements: clinical studies investigated the use of regional myocardial velocities in various adult conditions, such as ischemic heart disease, aortic regurgitation, and hypertrophic cardiomyopathy [7]. In an adult population with aortic stenosis, the degree of reduction in longitudinal systolic LV TDI velocities was shown to be related to the degree of fibrosis in the LV and parameters of longitudinal systolic function were also predictive of outcome after aortic valve replacement [8]. Kiraly et al. [9] showed that, in children with aortic valve stenosis, peak systolic and early diastolic wall velocities in the four-chamber view were significantly reduced. As CHD frequently affects the RV, data on right ventricular TDI velocities have been published in several conditions. In the normal (nonhypertrophied) RV, there is predominantly longitudinal orientation of the RV myofibres, making quantification of longitudinal TDI velocities especially important when assessing both systolic and diastolic RV function. Good correlations between systolic velocities and RVEF were found in an adult population that included patients with congenital heart disease [10]. Eyskens et al. have shown elevated RV systolic velocities in patients with ASDs and dilated RVs before percutaneous closure of the defect, which normalized within 24 hours after closure [11]. Quantitative assessment of RV performance after repair of tetralogy of Fallot (TOF) has also been investigated. TDI velocities were found to be decreased in TOF patients after surgical repair [12]. All TOF patients had normal LV myocardial velocities in this study, while 48 patients had reversed myocardial velocities in diastole in the RV free wall [12].

The use of TDI velocities in a functionally univentricular heart has also been studied. Frommelt et al. [13] used these measurements for the evaluation of 17 patients with hypoplastic left heart syndrome and showed a nonsignificant trend towards a decrease in systolic and diastolic tissue velocities from the neonatal period until after the second stage palliation, with no difference regarding the type of initial palliation. Mechanical dyssynchrony, as assessed by, for example, TDI, is often present in patients with cardiomyopathy but unrelated to electrical dyssynchrony and correlates with the severity of LV dysfunction [14, 15]. Such mechanical dyssynchrony probably reflects regional differences in myocardial dysfunction. Mechanical dyssynchrony evaluated by TDI was analysed in only 64% of patients in a multicenter European study evaluating the current practice and results of cardiac resynchronization therapy in paediatric and CHD [16].

Applications of TDI for RV function analysis in pediatrics have also been investigated. In healthy neonates born at or near term, the longitudinal systolic tricuspid annular velocity was only 1.2 times that of the mitral annular velocity [17]. This ratio of TV/MV excursion velocity differs from adults, in whom tricuspid annular velocity far exceeds mitral annular velocity, and it is speculated that this discrepancy may be the result of the increased afterload faced by the neonatal versus the adult RV.

Color TDI was introduced as an alternative technique for measuring tissue velocities. In contrast to pulse wave (PW) Doppler, which measures peak velocities, color TDI uses autocorrelation techniques in order to measure regional mean velocities. An advantage of colour TDI versus PW TDI is that tissue velocities can be recorded simultaneously in different myocardial segments during the same cardiac cycle. Kukulski et al. [18] demonstrated that TDI is feasible for the assessment of RV function by using the colour TDI to measure regional velocities at the TV annular, basal, mid, and apical regions of the RV free wall in 32 healthy subjects. The authors found significant variability in RV systolic velocities in all myocardial regions among healthy subjects. The RV velocities were higher than those recorded in corresponding LV segments. Measurement of longitudinal RV function by TDI is outlined further below in a separate section.

The advantage of TDI in CHD is that the technology can be applied to any chamber morphology and variables are not based on assumption of chamber geometry. The limitations of PW TDI and color Doppler TDI are mainly related to the variability of the measured velocities with different loading conditions. This is often more of a problem when assessing the RV free wall than septal of LV free wall TDI velocities.

## 3. Myocardial Performance Index (Tei Index)

The myocardial performance index (Tei index) evaluates global ventricular function by measuring the ratio of isovolumic time intervals to ventricular ejection [19]. The longer the isovolumic phases, the higher the Tei index, and the worse the ventricular performance. While the Tei index was initially developed for the assessment of global LV function, Eidem et al. found the Tei index useful in evaluating RV function in patients with CHD [20]. In this study, patients with large atrial septal defects represented the clinical setting of increased ventricular preload, whereas patients with isolated pulmonary valve stenosis represented increased RV afterload. Patients with congenitally corrected transposition of the great arteries with severe left atrioventricular valve regurgitation represented a combined increase in RV preload and afterload. No significant change in the RV Tei index was seen in any postoperative patient group despite relief of RV volume or pressure overload [20].

In the neonatal period, the RV and LV TEI were found to inversely correlate with RV output and EF, respectively, while the LV Tei index was found to correlate inversely with LV output and LV EF in the neonatal period as assessed using pulsed-Doppler echocardiography [21]. In addition, Tei indices for both RV and LV were shown to be significantly impaired in asphyxiated neonates [22]. The Tei index has also been reported to predict the outcome in children with heart failure [23]. Tissue velocity tracings have also been used to calculate Tei index, with significant correlations with PW Doppler measurements [24]. At the lateral and septal site, the Tei index measured by TD correlated well with conventional Tei index, both in healthy subjects and patients with dilated CMP. The highest correlation was observed in mean values of Tei index by TD (*r* = 0.94) in healthy subjects in this study [24]. The use of TD imaging is also useful for the RV, allowing simultaneous measurement of systolic and diastolic velocities indicative of cardiac systolic and diastolic function. The RV Tei index has been described as being useful in the assessment of 15 patients after repair of TOF and other forms of CHD [25]. In this study, the Tei index obtained by the pulsed Doppler method in TOF patients did not differ from that in normal children (0.30 ± 0.12 versus 0.32 ± 0.07). TDI showed that TOF patients had significantly decreased E, A, and S velocities compared to those of normal children. They also show that the Tei index, as measured by TDI, was significantly greater in TOF patients than in normal children (0.48 ± 0.07 versus 0.30 ± 0.07) [25]. The RV Tei index correlated well with pulmonary artery pressure [26] and has been used to identify early RV dysfunction in CHD [20]. Roberson and Cui investigated the RV Tei index in children and adolescents. The RV Tei index was calculated in this study by both TDI and PW-Doppler in 308 children. RV Tei index normal value as measured by TDI was 0.37 ± 0.05, and the RV Tei index normal value as measured by PW-Doppler was 0.34 ± 0.06 [27]. Cheung et al. showed that the Tei index is affected significantly by acute changes in loading conditions and was unable to consistently detect acute changes in LV contractile function by calculating the Tei index [28], making its interpretation difficult in the clinical settings.

TDI-Tei index (TDI-MPI) differs from the conventional Tei index in that it is measured in a single cardiac cycle, increasing its accuracy. The TDI-Tei has also been used to assess RV function in patients with pulmonary regurgitation (PR) after TOF repair and was significantly higher (=impaired) in TOF patients compared with controls, while there was no difference in conventional Tei index between TOF patients and control subjects [25]. In postoperative TOF patients, others have reported a good correlation of the Tei index with RVEF measured by cardiac MRI [29]. The advantage of the TEI index is that it is a combined ventricular-vascular index and fairly independent of ventricular geometry.

Limitations of the Tei index include the combination of systolic and diastolic time intervals in a single index which does not allow distinction between systolic and diastolic dysfunction (when the TEI index is abnormal, an appropriate interpretation is “abnormal systolic or diastolic function”). An additional limitation of the RV (versus LV) Tei index is that the Doppler beam cannot be aligned in a way that it runs through the RV inflow and RV outflow, which is possible for the mitral inflow and LVOT. Therefore, for the RV Tei index, two traces need to be recorded at identical heart rates, which may be impractical in the clinical setting. Caution is warranted when interpreting RV Tei indices obtained at even slightly different heart rates.

## 4. Myocardial Acceleration during ****Isovolumic Acceleration (IVA)

The isovolumic acceleration assesses myocardial motion during the isovolumetric contraction period. During this time period, there is a myocardial motion, related to the shape change from a more spherical ventricle to a more ellipsoid shape. The rate of myocardial acceleration during the isovolumic period has been described to correlate with intrinsic myocardial contractility and is thought to be relatively independent of loading conditions [30]. IVA can be used to assess contractile reserve during regular exercise stress testing [31] and stress echocardiography [32]. IVA has also been validated as a sensitive noninvasive index of LV and RV contractility [33]. Normal adult values of IVA measured in the basal segment of the RV free wall are >1.1 m/s^2^. The heart-rate sensitivity of IVA has been used to assess contractile reserve by studying the force-frequency relationship during pacing, stress echocardiography, or exercise [32, 34]. In paediatric patients, LV IVA has been used after heart transplantation, where it was suggested to be a useful additional noninvasive marker of allograft rejection [35]. Vogel et al. [30] used tricuspid annulus IVA to assess the systemic RV in patients who had undergone an atrial switch procedure for d-TGA. The tricuspid IVA in this population was lower than the corresponding LV IVA. RV IVA was also shown to be decreased and inversely related to the degree of PR in patients with repaired TOF, suggesting reduced contractile RV function in these patients. RV systolic dysfunction in patients with mitral stenosis could also be detected early using this technique [34], and Cheung et al. described a preserved systolic contractile reserve in children with single ventricle physiology after Fontan operation [36].

The advantage of IVA is that it is a relatively load-independent measure of RV function. A limitation of IVA is that it is sensitive to heart rate and carries—according to our experience—a relatively high interobserver variability.

## 5. Deformation Imaging

Wall motion abnormalities are relatively common in patients with CHD. Strain and strain rate seem to be useful in detecting such motion abnormalities. Regional deformation (strain) and strain rate can be calculated noninvasively in both the LV and RV, providing information on regional myocardial dysfunction in a variety of clinical settings. Myocardial velocities and displacement are influenced by global cardiac translational motion and by motion in adjacent myocardial segments (myocardial tethering) which limits their use in the assessment of regional myocardial function. This limitation can be overcome by using regional myocardial deformation or strain imaging.

*Myocardial strainand strain rate* are measures of deformation within segments of the myocardium and provide additional measurements of myocardial mechanical function independent from myocardial velocity. Strain is expressed as the percentage change in length from the original length. *Strain rate* is the rate of deformation (per second). Assessment of values from several segments can be done simultaneously. Strain imaging has been shown to be able to quantify RV function in patients with CHD [37] and to detect subclinical myocardial dysfunction both in patients receiving anthracyclines [38] and in young patients with muscular dystrophy [39]. Weidemann et al. first published deformation values in healthy children [40]. Deformation imaging has also been performed in healthy neonates [41–43] and in asphyxiated neonates [44].

Two different technologies are currently available for studying regional myocardial deformation. The first technique is based on TDI and calculation of myocardial velocity gradients. The second is based on tracking speckles on the grey-scale images from frame to frame throughout the cardiac cycle and calculating the displacement of the speckles throughout the cardiac cycle (speckle tracking).

TD-derived deformation imaging is based on assessment of differences in tissue velocities within myocardial segments and reflects either lengthening or shortening of the segment in the longitudinal, radial, or circumferential dimension. The strain rate is calculated as the velocity difference between two or more points within the myocardial segment divided by the distance between them, assessing the velocity gradient within the segment. Such velocity gradients actually quantify the instant regional deformation within the segment, expressed as the strain rate. The temporal resolution is high, and higher than for two-dimensional speckle tracking techniques and for MRI. The disadvantage of TDI-derived deformation imaging is that it is angle dependent and requires postprocessing [45]. Measurements are prone to disturbances from poor image quality, and the reproducibility of the measurements can be low. Despite these limitations, radial strain evaluation in the LV posterior wall has been suggested as a reproducible technique that performs better than speckle-tracking techniques [45]. Longitudinal strain measurements in the interventricular septum and LV and RV lateral walls have been used to quantify regional and global myocardial function. LV ejection fraction is generally preserved in patients with hypertrophic cardiomyopathy (HCM), but deformation imaging has demonstrated significant regional differences in systolic deformation variables. Peak systolic strain was shown to be reduced significantly in the more hypertrophic regions of HCM patients [46, 47]. In young patients with Duchenne muscular dystrophy, deformation analysis showed a significant decrease in radial and longitudinal peak systolic strain and strain rate in the LV inferolateral wall in patients with normal EF [48]. In patients studied during follow-up after anthracycline exposure, a decrease in deformation variables was observed, despite normal EF and fractional shortening [49]. Eyskens et al. demonstrated that TOF patients had decreased peak systolic strain and strain rate values in the basal, mid, and apical segments of the RV free wall and interventricular septum, which was related to the degree of pulmonary regurgitation [50]. Reduced regional peak systolic strain and strain rate values in the apical segments of the RV free wall were also found in CHD patients with a systemic RV [37]. Bos et al., described reduced longitudinal RV deformation in patients with congenitally corrected transposition of the great arteries [51]. 

Speckle tracking is based on grey-scale images and is relatively easy to perform. In contrast to TDI-derived parameters, speckle tracking is an angle-independent technique as the movement of speckles can be followed in any direction within the 2D grey scale image. As the method is based on recognition of speckles between frames, there are upper and lower limits for the frame rate. The frame rate has to be high enough so that speckles are recognizable between frames; if the frame rate is too low, then the speckles might change too much between frames or the speckles might not be recognized due to movement out of the plane. The temporal resolution is low compared to TDI, which might be an issue especially in smaller children with higher heart rates. Current two-dimensional longitudinal deformation analysis is to large extent based on assessment of the AV-valve plane motion. Regional differences might therefore not be detected, but, at the same time, the global values might be less influenced by local or regional artefacts. Software from different vendors can yield different results [45]. Good correlation between two-dimensional speckle strain and MRI tagging for the LV and RVs has been reported [52, 53]. The major clinical applications of speckle-tracking techniques should be the same as for the TDI-derived techniques. As described recently, the major advantage of speckle-tracking technology is that it allows the study of radial, longitudinal, and circumferential deformation as well as the assessment of ventricular rotation and torsion [54]. Kowalski et al. showed decreased deformation in the LV anterior wall after successful repair of aortic coarctation [55]. Laser et al. showed that, in patients with aortic stenosis and aortic coarctation, LV torsion was increased before and decreased after interventional treatment [56]. Dragulescu and Mertens have nicely reviewed that speckle-tracking techniques can be used in children to reliably quantify longitudinal and circumferential strain [57]. Left ventricular torsion seems to be impaired in conditions associated with RV volume load, mostly due to reduced basal rotation. In young adults, acute unloading of the RV after transcatheter closure of a secundum ASD improves LV twist by increasing basal rotation [58]. Cheung et al. have shown that RV dilatation has a negative impact on LV circumferential deformation but not longitudinal or radial deformation [59]. Kutty et al. investigated the changes in RV function in patients after TOF repair after surgical pulmonary valve replacement [60]. A significant increase in peak systolic velocities but not in global longitudinal strain was seen, and all indices remained significantly lower in TOF patients compared with normal values. A recent study evaluated the acute effect of transcatheter pulmonary valve implantation, demonstrating an improvement in RV free wall and septal longitudinal function [61].

### 5.1. Comparison of TDI and 2D-Based Deformation Imaging

Tissue velocities and strain measurements have been shown to be useful to quantify global and regional RV function in adult pulmonary arterial hypertension (PAH) patients. The more severe the PAH, the lower the end-systolic longitudinal strain in the RV free wall [62, 63], demonstrating a dependence of systolic RV longitudinal strain measurement on RV afterload [64, 65]. In a comparison of TDI and 2D speckle tracking, comparable values were found for speckle tracking and TDI in both normal and dysfunctional segments and in radial measurements, but 2D speckle tracking was suggested to be more reliable than TDI [66]. A disadvantage of two-dimensional speckle tracking is that it has a relative low temporal resolution that hinders tracking in the presence of high heart rates [67]. Postprocessing time was shown by Ingul et al. to be significantly shorter with speckle tracking compared to TDI analysis [68]. Teske et al. show that the quantification of regional RV function using either 2D speckle tracking or TDI is feasible and reproducible [53, 69]. Strain and strain rate might be relatively independent of loading conditions and may be preferred over tissue velocities as they are not influenced by passive motion of the myocardium when the heart moves within the chest. The future ease of using 2D-based deformation imaging techniques with limited postprocessing times will probably results in a more routine application of this technique, but at present the different solutions offered by the competitive software vendors remain a major challenge, and the need for an industry standard for these measurements is needed [45].

### 5.2. Cardiac Magnetic Resonance Imaging-Derived Tissue Tracking

Tissue tracking of cardiac magnetic resonance imaging (CMR) is a novel method with only little published research on this topic [70–73]. Niemann et al. introduced a speckle-tracking program, the velocity vector imaging applied to CMR imaging in normal hearts [70]. In patients with a single ventricle, a decrease in apical rotation and circumferential strain compared to controls were found [71]. Authors concluded that speckle tracking applied to CMR imaging sequences was able to detect reduced strain and rotational motion in patients with single ventricle. For a detailed description of this novel method, we refer to Truong et al. [71]. Ortega et al. [72] investigated the impact of LV dyssynchrony on clinical outcomes of TOF patients. They quantified LV dyssynchrony as the maximum difference in time to peak radial displacement, circumferential strain, and radial strain among ventricular segments and furthermore the standard deviation of the times to peak value. In this study [72], no attempt was made to study the mechanics of the RV or the impact of ventricular interactions because the application of tissue tracking to the RV has not been validated to date. A limitation is that speckle tracking applied to CMR is based on gray-scale imaging and is therefore dependent on the quality of the images. At present, the time resolution is much lower than for both the ultrasound techniques. Higher frames rate (>30 frames/s) would also improve tracking and accuracy of the measurements.

## 6. Three-Dimensional Echocardiography 

Three-dimensional (3D) echocardiographic rendering of cardiac structures of patients with CHD has gained interest during recent years with the development of high-frequency paediatric probes with improved image quality [74]. Using these probes, full volumetric datasets of the beating heart can be obtained which can then be cropped to analyze cardiac anatomy. The most common method of assessing RV volume uses semiautomated border detection and a model of the RV that is used in semiautomated RV volume reconstruction. This technique has been described to be accurate in patients with CHD [75, 76]. With the newer generation of echocardiography machines, end-diastolic volume calculations of 3D datasets are available, with good correlations with other techniques such as the MRI [77–79]. 3D echocardiography is dependent on adequate acoustic windows. Khoo et al. focussed on determination of RV volumes in adolescents and young adults with CHD [80]. This study showed that, using different quantification techniques, 3D measurements were only feasible in about 50% of all patients [80], due to inadequate image quality. Although the authors only used “good” imaging data sets for their analysis, they showed about 20 percent lower RV volume values when compared to parallel MRI measurements [80]. A similar underestimation of the RV volume when compared to MRI measurements was reported from a different group that found the underestimation of RV volumes by 3D echocardiography mainly occurring in severely dilated RVs [81]. Accurate measurement of RV volumes is important for certain diseases such as postoperative TOF patients, where RV end-diastolic volume index is an important variable in the decision for the need of a pulmonary valve replacement. Three-dimensional echocardiography has also been studied in various CHD lesions [82–84]. The addition of 3D color-Doppler imaging can be helpful in planning a detailed preoperative assessment. The challenge will be how this can be integrated into daily clinical practice. In patients with PAH, a good correlation was found in the measurements of RVEF (*r* = 0.66) and RVEDV (*r* = 0.74) determined by either 3D echocardiography or MRI measurements [85]. The use of 3D has been validated for the measurement of RV volumes and EF, which correlated well with those variables determined by CMR imaging in adult and pediatric populations [86, 87]. Moreover, Kjaergaard et al. reported the use of 3D echocardiography for the evaluation of RV cardiomyopathy [88]. Normal values for right ventricular size and function in adults have been published [89]. 3D echocardiography outperformed 2D echocardiography in the assessment of RV volumes and compared favourably with cardiac MRI [90]. Validation of 3D echocardiographic assessment of LV volumes and LVEF in children with complex congenital heart disease including a small LV has been performed [91]. Soriano et al. have assessed volume and ejection fraction in pediatric patients with a functional single ventricle using 3D echocardiography [92]. The 3D echocardiographic end-diastolic volume correlated well but was smaller volume by CMR (by 9%), and EF by 3D echocardiography was smaller than EF by CMR (by 11%). There was no significant difference between end-systolic volumes and mass [92].

## 7. Systolic to Diastolic Ratio

**Figure 3:**
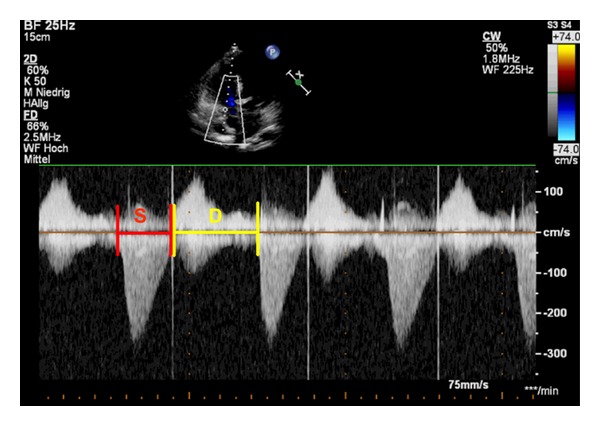
Apical 4-chamber view. Doppler-derived tricuspidal regurgitation time (TR) interval ratio of systolic (S) duration to diastolic (D) duration (S/D). The ratio of S/D duration was calculated by dividing the duration of the TR spectral Doppler flow pattern by the time interval of the cardiac cycle that did not include TR. Systolic and diastolic duration using TR duration was measured by CW-Doppler from the apical 4-chamber view to calculate the S/D ratio. The red lines show the systolic duration, and the yellow lines show the diastolic duration.

Friedberg and Silverman described the Doppler-derived mitral regurgitation time interval ratio of systolic (S) duration to diastolic (D) duration (S/D) [93]. The S/D ratio was found to be abnormally increased in children with dilated and restrictive cardiomyopathy [93, 94]. The ratio of S/D duration was calculated by dividing the duration of the mitral regurgitation (MR) spectral Doppler flow pattern by the time interval of the cardiac cycle that did not include MR [93]. Tei index and S/D ratio were shown to occasionally lack sensitivity and have a low negative predictive power due to a significant false-negative rate [95]. Friedberg and Silverman [96] investigated the S/D duration ratio in children with HLHS as a novel promising index of global RV function. They measured systolic and diastolic duration using tricuspid regurgitation (TR) duration by CW Doppler from the apical 4 chambers to calculate the S/D ratio in children with HLHS. Heart rate has been shown to be a major determinant of D and S duration and at resting heart rates, systole (S) constitutes about 40% of the cardiac cycle in healthy children [96], as shown in Figure 3. In patients with PAH, RV contraction is prolonged, although RV ejection time is shortened, so that S/D duration was proposed as an indicator of PAH severity [97]. Children with significant PAH were found to have a marked decrease in diastolic duration and increase of the S/D duration ratio when their heart rate increases compared to matched control subjects [98]. Alkon et al. found that an increased S/D duration ratio >1.4 inversely correlates with survival in children with PAH [98]. They concluded that, in children with PAH, an increased S/D ratio is temporally associated with worse RV function, hemodynamics, exercise capability, and survival.

Limitation of S/D duration ratio is that the measurement of this ratio to assess ventricular function requires the presence of defined onset and end of MR/TR on spectral Doppler tracings.

## 8. Measurement of Longitudinal RV Function 

**Figure 4:**
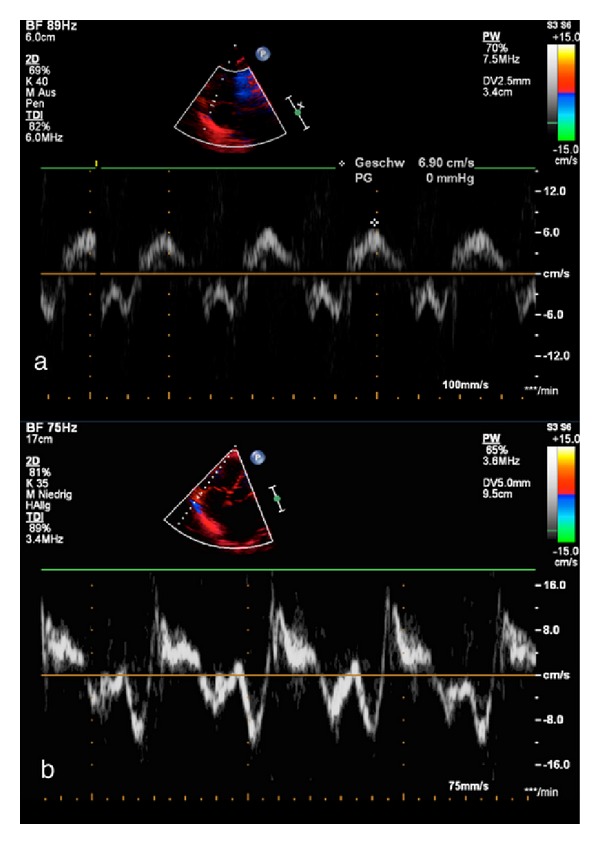
Apical 4-chamber view. The white broken line indicates M-mode cursor placement at the tricuspid lateral annulus. Representative image of the tricuspid annular peak systolic velocity (TAPSV) in a neonate (Figure 1(a)), and in a 15-year-old adolescent (Figure 1(b)), respectively, with normal right and left ventricular function.

**Figure 5:**
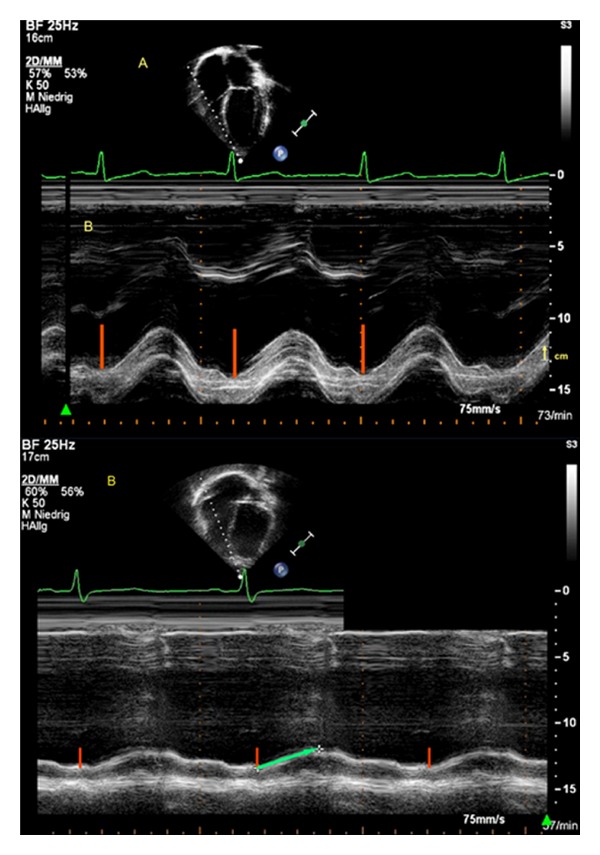
Apical 4-chamber view. (a) The white broken line indicates M-mode cursor placement at the tricuspid lateral annulus. Representative M-mode image of the tricuspid annular plane systolic excursion (TAPSE) in a patient with normal right and left ventricular function. The absolute longitudinal displacement measure is shown as the red line. The yellow arrow marks the upper and lower measure point of one centimeter (cm). (b) Representative M-mode image of the tricuspid annular plane systolic excursion (TAPSE) in a 17-year-old patient with TOF and a decreased TAPSE. The absolute longitudinal displacement measure is shown as the red line. The green arrow shows the decreased TAPSE value and flat course of the excursion.

Sheehan and Redington recently reviewed that the RV differs from the LV in its physiology with the LV output being less sensitive to afterload changes than the RV [2]. They suggest that this might be able to explain the fact that PAH is less tolerated compared to systemic arterial hypertension [2]. Echocardiographic assessment of RV function is commonly known to be technically difficult in both children and adults. The anterior position of the RV in the chest limits the echocardiographic visualization of the RV. Moreover, the RV has a complex geometry, with a triangular shape in the sagittal and a more crescent shape in the coronal plane. Furthermore, the RV inflow and outflow tracts are difficult to image simultaneously with 2-dimensional echocardiographic methods. In a normal RV, most of the myocytes are orientated longitudinally, which results in a different contraction pattern when compared to the LV [99]. Indices for assessment of longitudinal RV function have been published, including tricuspid annular plane systolic excursion (TAPSE) [100] and tricuspid annular peak systolic velocity (TAPSV) [101] (Figures 4 and 5). Measurement of myocardial velocities by TDI is a promising approach for quantitative assessment of longitudinal systolic ventricular performance [102]. TDI indicates systolic ventricular performance by measuring velocities directly from myocardium. By employing pulsed wave (PW)-TDI to the tricuspid annulus, it is possible to measure its peak systolic velocity [103]. The TAPSV, measured by PW-TDI, has been suggested as a good quantitative parameter of RV systolic function in adults [104] and children [105].

**Figure 6:**
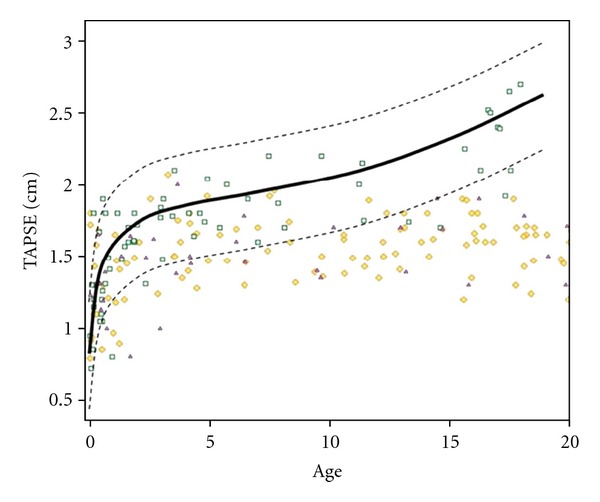
Course of TAPSE values in patients with PAH, PS, and TOF from mean reference values versus increasing time period. The TAPSE value data points for PAH, PS, and TOF patients are given as yellow oblique squares, green squares, and purple triangles, respectively. The interpolated mean values of control group are given as the black thin line. The −2 standard deviation line (−2SD) of the control group measurements is given as a grey dashed line. The difference of mean TAPSE values from mean reference values is expressed in centimetre (cm). PS: pulmonary stenosis; PAH: pulmonary artery hypertension; SD: standard deviation; TAPSE: tricuspid annular plane systolic excursion; TOF: tetralogy of Fallot.

**Figure 7:**
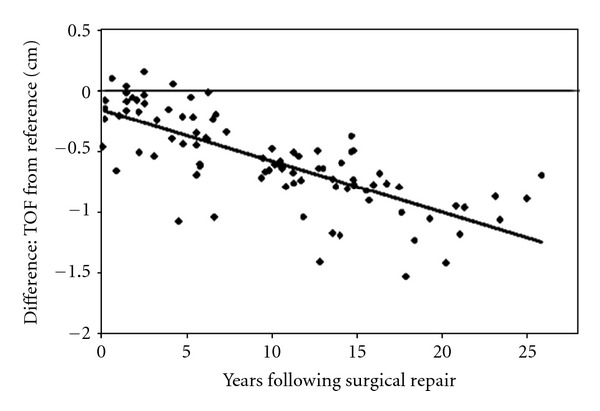
Deviation of TAPSE values in TOF patients from reference values versus years following surgical repair. The TAPSE value data points are given as black dots. Shown is the absolute deviation of the measured TAPSE values of TOF patients compared to TAPSE reference values. The difference of TAPSE values is expressed in centimetre (cm). The black solid line is an interpolation for a linear trend for the TOF patients. TAPSE: tricuspid annular plane systolic excursion; TOF: tetralogy of Fallot.

**Figure 8:**
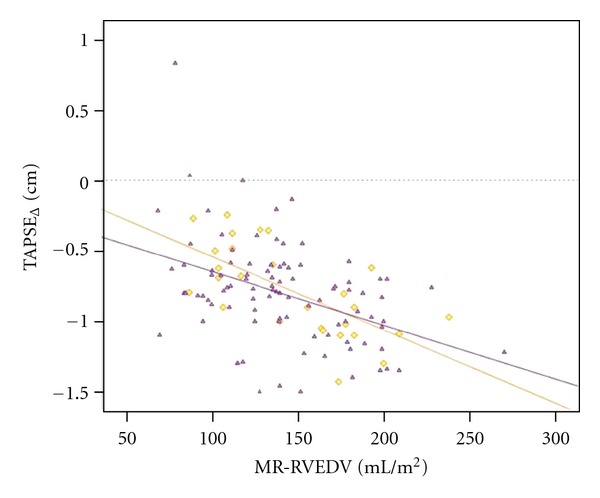
Relationship between TAPSE values and RVEDVi in PAH-CHD and TOF patients. TAPSE value data are given as yellow oblique squares for PAH-CHD patients and purple triangles for TOF patients, respectively. Deviation of TAPSE values in patients with PAH-CHD and TOF from mean age-related reference values is shown. The difference of TAPSE values to reference values is expressed in centimetre (cm). The yellow and the purple solid lines are an interpolation for a linear trend in PAH-CHD and TOF patients, respectively. PAH-CHD: pulmonary artery hypertension secondary to congenital heart disease; RVEDVi: indexed right ventricular end-diastolic volume; MR: magnetic resonance; TAPSE: tricuspid annular plane systolic excursion; TOF: tetralogy of Fallot.

The TAPSE is an indicator of ventricular contractile function that correlates well with RVEF in adults [107] and can also be used as an index of systolic RV function in pediatric patients with TOF and ASD [108, 109]. Reference values of TAPSE measurements in adults and across the full pediatric age range are available [106, 110]. In recently published recommendations for the performance of a pediatric echocardiogram, the value of TAPSE measurement is discussed in detail and recommended as additional tool for the investigation of longitudinal RV function [111]. Studies of PW-TDI measurements of the tricuspid annular velocity have been reported in children [6, 105, 112] and report conflicting data regarding the impact of age on TAPSV in pediatric patients. This may be due to small sample sizes, limited age distribution, or somewhat arbitrary age groups. Focussing on the TDI velocities, Roberson et al. [112] provide normal values of systolic S wave of tricuspid annulus versus age in the pediatric age group. However, this study reports a wide range of normal values from infancy to adolescents. In contrast, adult studies show that a TAPSV below 10.5 cm/s [104] is able to predict an impaired RV systolic function. Pediatric reference values of TAPSV normal data are available [106] as shown in Table 1. A significant positive correlation between TAPSV and TAPSE has been shown in adults with PAH (*r* = 0.90) [104]. Data have shown that TAPSE is a reproducible index of RV systolic function in patients with PAH, and abnormal TAPSE (below 1.8 cm) had a high specificity for abnormal RV function in adults [113]. They show that for every 1 mm decrease in TAPSE, the unadjusted risk of death increased by 17% for PAH patients [113]. A significant amount of PAH in childhood is secondary to CHD [114]. New echocardiographic techniques have been published with the aim of providing a profound determination of pulmonary artery pressure [115]. An abnormally low TAPSE was associated with poor prognosis in adults with idiopathic PAH [113]. Adult studies describe the usefulness of TAPSE to diagnose RV systolic dysfunction [116, 117]; it has been shown that a TAPSE <2 cm indicates a RVEF of <40% [118]. Recently, it has been reported that TAPSE measurement is a more reproducible indicator of RV function when compared to other echocardiographic variables such as the RV fractional area change [113]. TAPSE appears to be developmentally dependent variable that increases from preterm infants to healthy adolescents [109, 119]. The mitral annular plane systolic excursion as a M-mode parameter of the longitudinal LV systolic function has also been shown to be developmentally dependent variable that increases from infants to healthy adolescents [120]. In an ongoing investigation, it has been shown that with increasing age of PAH patients, that is, the longer the RV is exposed to severe pressure overload and potentially other conditions and environmental factors associated with PAH, TAPSE values become significantly decreased compared to age-matched controls [121] as shown in Figure 6. Despite the difficulty in determining the exact onset of PAH in the individual patient, this may point towards an early decline of RV systolic function. After a mean of 6.1 years after surgical correction for TOF, TAPSE values become significantly decreased and abnormal (>2 SD of age-matched controls), indicating reduced RV systolic function that is likely due to volume overload caused by significant PR [109], as shown in Figure 7. This observation is in agreement with a study showing RV systolic dysfunction in adult TOF patients [122]. In TOF patients with a decreased TAPSE (*z*-score < −2 SD), an inverse and significant correlation was found between TAPSE and RVEF measured by MRI in 88 children (*r* = 0.54) [100] and in adults (*r* = 0.48) [123]. The correlation between RVEDVi and TAPSE was significantly negative (*r* = −0.42; *P* < 0.001) in TOF and PAH-associated with congenital heart disease (CHD) patients as shown in Figure 8. The latter finding is surprising given common idiopathic PAH knowledge, but the patients in this study suffer from a PAH-CHD after heart surgery in early childhood, and therefore a difference to idiopathic PAH data is to be expected [121].

One limitation is that, although TAPSE appears to be a good indicator of global RV function, it does not take into account segmental RV function and contractility. A major limitation to any novel assessment of RV systolic function is the lack of an acceptable quantitative echocardiography standard of comparison.

## 9. Perspectives 

The future of newer echocardiographic techniques, such as tissue Doppler imaging (TDI), deformation imaging (tissue tracking), myocardial acceleration during isovolumic contraction (IVA), S/D duration ratio, 3D echocardiography, and measurements of systolic RV function, depends on further validation and demonstration of clinical utility. There is still a long way to go before relevant clinical decisions can be based solely on these new echocardiographic measures. In particular, prospective comparative studies and well-designed randomized controlled trials with such variables as primary and secondary outcomes are desirable. However, the new imaging methodologies already offer new insights into the effects of congenital heart disease on LV and RV function and mechanics. The assessment of ventricular function by echocardiography is an area that is undergoing intensive research, as the development and implementation of new modalities become available. Cardiac sonographers should become familiar with both traditional and newer techniques such as tissue Doppler imaging, deformation imaging, S/D duration ratio, 3D echocardiography, and measurements of systolic RV function, for more detailed assessments of ventricular performance.

## Figures and Tables

**Table 1:** Classification table for the TAPSV values of our healthy individuals. For the purpose of the study only echocardiograms with an official reading of completely normal study were accepted for analysis. The values in the classification table are shown as follows. For each age group the standard deviation (SD) of TAPSV values was taken to construct ranges of the mean ± 2SD and ±3SD. These ranges represent the expectable normal intervals of deviation for a certainty level of 95% and 99%. Abbreviations: BSA, body surface area; SD, standard deviation; TAPSV, tricuspid annular peak systolic velocity. Copyright from Koestenberger et al. [106] with permission from the publisher, Copyright 2009, the Journal of the American Society of Echocardiography.

1st month	83	7,2	4,8	9,5	3,6	10,7	0,22	0,14	0,34
2nd month	34	8,5	6,5	10,5	5,5	11,5	0,25	0,22	0,31
3rd month	18	8,7	6,3	11	5,1	12,2	0,27	0,19	0,33
4th month	28	9,1	6,3	11,8	4,9	13,2	0,29	0,19	0,37
5th month	11	9,8	6,4	13,2	4,7	14,9	0,32	0,24	0,38
6th month	9	9,1	7,5	10,6	6,7	11,4	0,31	0,27	0,4
7th month	18	9,5	7,3	11,8	6,1	12,9	0,35	0,28	0,41
8th month	8	9,7	6,4	12,9	4,7	14,6	0,37	0,32	0,45
9th month	9	9,9	6,4	13,4	4,7	15,1	0,39	0,34	0,44
10th month	13	10,6	8,1	13,1	6,9	14,4	0,4	0,28	0,48
11th month	11	11,1	8,1	14,1	6,6	15,6	0,37	0,24	0,47
12th month	13	11	7,7	14,4	6	16,1	0,39	0,3	0,47
2nd year	55	11,4	8,7	14	7,4	15,4	0,51	0,37	1,02
3rd year	34	11,7	8,3	15,1	6,6	16,9	0,58	0,47	0,7
4th year	38	12,2	9,3	15	7,9	16,4	0,64	0,4	0,82
5th year	43	12,3	9,4	15,2	8	16,6	0,72	0,56	0,84
6th year	43	12,4	9,6	15,3	8,2	16,7	0,79	0,67	1
7th year	33	12,6	9,7	15,4	8,3	16,8	0,87	0,67	1,18
8th year	40	12,7	9,8	15,6	8,3	17	0,95	0,74	1,39
9 th year	25	12,5	9,5	15,5	8	17,1	1,02	0,77	1,47
10th year	23	12,8	10,4	15,2	9,2	16,4	1,22	1,08	1,47
11th year	28	13,1	10,3	15,9	9	17,3	1,31	1	2
12th year	33	12,9	9,9	16,4	7,6	18,2	1,42	1,03	1,75
13th year	25	13,2	10,7	15,8	9,4	17,1	1,51	1,06	1,87
14th year	29	13,3	10	17,7	6,6	19,9	1,57	0,83	1,98
15th year	37	13,8	10,5	17,1	8,9	18,8	1,66	1,37	2,07
16th year	37	14,1	10,1	18,1	8,1	20,1	1,7	1,3	2,06
17th year	43	14	10,1	17,9	8,2	19,8	139	1,45	2,3
18th year	39	14,3	10,7	17,9	8,9	19,8	1,71	1,4	2,05

## Abbreviations

ASD: Atrial septal defect
CHD: Congenital heart disease
CMP: Cardiomyopathy
EF: Ejection fraction
IVA: Isovolumic acceleration
LV: Left ventricle
MR: Mitral regurgitation
MRI: Magnetic resonance imaging
PAH: Pulmonary artery hypertension
PR: Pulmonary regurgitation
QRSd: QRS Duration
RV: Right ventricle
RVEDVi: Indexed RV end-diastolic volume
S/D duration: Systolic to diastolic duration
TAPSE: Tricuspid annular plane systolic excursion
TAPSV: Tricuspid annular peak systolic velocity
TDI: Tissue Doppler imaging
TGA: Transposition of the great arteries
TOF: Tetralogy of Fallot
TR: Tricuspid regurgitation
TTE: Transthoracic echocardiography
2D: Two-dimensional
3D: Three-dimensional.
